# The association between eicosanoids and incident atrial fibrillation in the Framingham Heart Study

**DOI:** 10.1038/s41598-022-21786-0

**Published:** 2022-11-23

**Authors:** Jelena Kornej, Maha A. Qadan, Mona Alotaibi, David R. Van Wagoner, Jeramie D. Watrous, Ludovic Trinquart, Sarah R. Preis, Darae Ko, Mohit Jain, Emelia J. Benjamin, Susan Cheng, Honghuang Lin

**Affiliations:** 1 National Heart, Lung, and Blood Institute, Boston University’s Framingham Heart Study, Framingham, MA USA; 2 Section of Cardiovascular Medicine, Department of Medicine, Boston Medical Center, Boston University School of Medicine, Boston, MA USA; 3 Department of Cardiovascular and Metabolic Sciences, Lerner Research Institute, Cleveland Clinic Foundation, Cleveland, OH USA; 4 Division of Pulmonary, Critical Care and Sleep Medicine, University of California San Diego, La Jolla, CA USA; 5 Department of Medicine, University of California, La Jolla, San Diego, CA USA; 6 Department of Biostatistics, Boston University School of Public Health, Boston, MA USA; 7 Department of Epidemiology, Boston University School of Public Health, Boston, MA USA; 8 Department of Cardiology, Cedars-Sinai Medical Center, Smidt Heart Institute, Los Angeles, CA USA; 9 Department of Medicine, University of Massachusetts Chan Medical School, Worcester, MA USA

**Keywords:** Biomarkers, Cardiology, Medical research

## Abstract

Chronic inflammation is a continuous low-grade activation of the systemic immune response. Whereas downstream inflammatory markers are associated with atrial fibrillation (AF), upstream inflammatory effectors including eicosanoids are less studied. To examine the association between eicosanoids and incident AF. We used a liquid chromatography-mass spectrometry for the non-targeted measurement of 161 eicosanoids and eicosanoid-related metabolites in the Framingham Heart Study. The association of each eicosanoid and incident AF was assessed using Cox proportional hazards models and adjusted for AF risk factors, including age, sex, height, weight, systolic/diastolic blood pressure, current smoking, antihypertensive medication, diabetes, history of myocardial infarction and heart failure. False discovery rate (FDR) was used to adjust for multiple testing. Eicosanoids with FDR < 0.05 were considered significant. In total, 2676 AF-free individuals (mean age 66 ± 9 years, 56% females) were followed for mean 10.8 ± 3.4 years; 351 participants developed incident AF. Six eicosanoids were associated with incident AF after adjusting for multiple testing (FDR < 0.05). A joint score was built from the top eicosanoids weighted by their effect sizes, which was associated with incident AF (HR = 2.72, CI = 1.71–4.31, *P* = 2.1 × 10^–5^). In conclusion, six eicosanoids were associated with incident AF after adjusting for clinical risk factors for AF.

## Introduction

Increased global life expectancy and longer survival with chronic conditions, including atrial fibrillation (AF), have prompted a keen interest in preventing or postponing age-related common chronic diseases and preserving wellness in the population^1–3^. Chronic inflammation is a continuous low-grade activation of the systemic immune response. Inflammation is a major feature of biological aging across multiple organ systems, including the cardiovascular system. Also, inflammation has been associated with “accelerated aging” phenotypes and reduced lifespan^4,5^. The majority of research analyzing inflammation has focused on *downstream* markers of inflammatory activity such as cytokines (e.g., tumor necrosis factors, interferons) and acute phase reactants (e.g., C-reactive protein, interleukins)^6^. Chronic elevation of downstream markers is associated with multiple age-related disease states and shorter lifespan^7^. However, evidence for a clinically important, causal role of these biomarkers is inconsistent^8,9^. It has been suggested that causative actors in the inflammatory arena are more likely to be *upstream* effectors^10^.

Data from small studies suggest that select eicosanoids are related to risk factors such as obesity^11^, diabetes^12^, and smoking^13,14^. Eicosanoids are biologically active lipid mediators derived from 20-carbon polyunsaturated fatty acids (PUFAs)^15^. Eicosanoids have significant activities in the regulation of normal physiological processes and disease pathogenesis in the human body^16,17^. Eicosanoids have pleiotropic roles in inflammation and immunity^18,19^. Many studies have focused on strategies for inhibiting the formation of inflammatory mediators that may contribute to risk of AF^20^. In contrast, epoxyeicosatrienoic acids act mainly as autocrine and paracrine effectors in the cardiovascular system and kidney, mediating vasorelaxation, anti-inflammatory, and pro-fibrinolytic processes, as well as several cardiovascular protective effects^21^. Therefore, blocking the process of metabolizing epoxyeicosatrienoic acids into diverse pro-inflammatory compounds (e.g., 1,2-diols, dihydroxyeicosatrienoic acids), leads to increases in the titers of epoxyeicosatrienoic acids, which in turn may contribute to the prevention of AF through attenuation of atrial structure and electrical remodeling^22–24^.

However, the role of eicosanoids as upstream biomarkers of incident AF is largely unknown. We hypothesized that increased plasma levels of eicosanoids are associated with increased rates of incident AF in the community.

## Methods

All data and materials are available at the National Heart, Lung, and Blood Institute Biologic Specimen and Data Repository Information Coordinating Center and can be requested at https://biolincc.nhlbi.nih.gov/studies/framcohort/. All procedures performed in studies involving human participants were in accordance with the ethical standards of the institutional and/or national research committee and with the 1964 Helsinki Declaration and its later amendments or comparable ethical standards. The Framingham Heart Study protocol was approved by the Boston University Medical Center Institutional Review Board (Approval Number H-32132) and all participants (or proxies) signed informed consent.

### Study population

We analyzed data from the Framingham Heart Study (FHS). The FHS Original cohort (n = 5209) was established in 1948 to investigate risk factors for cardiovascular disease^25^. In the early 1970s, the children of the Original cohort participants and their spouses were enrolled into the Offspring cohort (n = 5124)^26^. A study sample (n = 2676 participants) was drawn from the FHS Offspring cohort who attended exam eight (2005–2008, total n = 3021 exam attendees) with archived blood specimens available for eicosanoid profiling.

### Follow-up and covariate measurement

Participants in the Offspring cohort were routinely evaluated by history and physical examination in the research center by FHS clinicians approximately every 4–8 years. Participants were under surveillance during inter-exam periods for cardiovascular outcomes through review of outside medical records and clinician visits adjudicated by FHS investigators. Incident AF (atrial fibrillation and atrial flutter) was diagnosed if it occurred after the eighth examination by electrocardiogram at a FHS examination or if it was documented in the participants’ outside medical records, interim hospitalizations, and Holter monitor results. Prevalent AF cases, defined as those that were diagnosed with AF at or before their eighth clinical examination, were excluded from the analyses. Participants were followed from the date of their eighth FHS examination until the occurrence of AF, death, loss to follow-up, or December 31, 2018, whichever occurred first.

Age, sex, diabetes, blood pressure, self-reported smoking status, and antihypertensive medication use were recorded during each examination. Hospital records were reviewed to evaluate history of heart failure and myocardial infarction by 2–3 clinicians (the Framingham Endpoint Review Committee) between FHS follow-up exams prior to AF diagnosis. Heart failure was diagnosed based on the simultaneous presence of at least two major criteria or one major and two minor criteria as previously described^27^. History of myocardial infarction was designated if there were at least two of three findings: (1) symptoms indicative of ischemia; (2) changes in blood biomarkers of myocardial necrosis; (3) serial changes in electrocardiogram. Deaths were documented by death certificates. Additional information was obtained from records supplied by hospital, attending physician, pathologist, medical examiner, or family members.

### Measurement of eicosanoids

Details of eicosanoid profiling have been described in detail previously^28,29^. In brief, eicosanoids and eicosanoid-related metabolites were analyzed using liquid chromatography-mass spectrometry (LC–MS), using a Vanquish UPLC coupled to high resolution, Q Exactive Orbitrap mass spectrometer (Thermo Fisher, Waltham, MA, USA). Metabolites were measured using a Phenomenex Kinetex C18 column, for the measurement of 161 eicosanoids and eicosanoid-related metabolites. Each eicosanoid is represented by MZ/RT, in which MZ is the mass-to-charge ratio (to 5 decimals) and RT is the retention time (to 4 decimals). QC/QA analysis was performed, and spectral data were extracted as previously described. We imputed missing values with the minimum value for each eicosanoid. Metabolites measurements were log_e_ transformed and adjusted for age and sex. Residuals of the regressions were normalized using the median absolute deviation of each eicosanoid, as in a previous study^30^.

### Assay of Eicosanoid Markers of Inflammation

Eicosanoid biomarker assays were performed in the (University of California San Diego, Dr. Jain laboratory) by the use of mass spectrometry (MS) after separation of lipids by gas chromatography (GC) or liquid chromatography (LC). The MS infrastructure used in this study includes an AB SCIEX QTRAP 6500 MS interfaced with a Waters Acquity UPLC system, an AB SCIEX QTRTAP 4000 MS coupled to a Shimadzu HPLC system, and an Agilent 6890 N Gas Chromatograph equipped with an Agilent 5975 Mass Selective Detector. Eicosanoids (> 75 in total) were isolated by solid phase extraction and quantified by reverse phase LC–MS using electrospray ionization (ESI) and multiple reaction monitoring (MRM). The eicosanoid markers included prostaglandins (e.g. PGF1beta, PGF1alpha, PGG2), leukotrienes (e.g. 20-carboxy-LTB4), and clavulones (e.g. clavulone I). A sample of 150 µl of EDTA plasma was required for each participant per exam.

### Measurements of inflammation biomarkers

Blood samples were collected after an overnight fast and stored at − 80 °C until assayed. A detailed measurement protocol has been described previously^31^. CRP measurements were performed in serum using immunoturbidimetry (Roche Diagnostics Latex High Sensitivity Assay), and Interleukin 6 (IL-6) was analyzed using commercially available enzyme-linked immunosorbent assay kits (R&D Systems, Minneapolis, MN, USA) as previously described^32–34^. Both CRP and IL-6 concentrations were measured at the same examination when eicosanoids were profiled. Detailed information regarding biomarker assessments can be found at the FHS website: https://framinghamheartstudy.org/files/2017/08/Inflammatory-Biomarker-Protocol-Offspring-Exam-8-and-Omni-1-Exam-3.pdf.

### Statistical analysis

Descriptive statistics were calculated using means and standard deviations for continuous variables, or frequency counts and percentages for dichotomous variables. The association of each eicosanoid with incident AF was assessed using Cox proportional hazards models with robust sandwich estimators (to account for the relatedness of some participants), with follow-up times censored at the last follow-up time or death. Participants who were diagnosed with AF before examination 8 (prevalent AF, n = 210) were excluded from the Cox models. The primary models were adjusted for previously reported AF-related clinical factors^35^, including age, sex, height, weight, systolic and diastolic blood pressure, current smoking, use of antihypertensive medication, diabetes, history of myocardial infarction, and history of heart failure. The proportional hazard assumption was examined using visual analyses of the curves and Schoenfeld’s test^36^.

We further categorized eicosanoids into three tertile groups and examined their difference in terms of incident AF risk. In the exploratory models, we additionally adjusted for the concentration of CRP and IL-6 to understand the influence of downstream inflammatory biomarkers on the association between eicosanoids and incident AF.

We adjusted for multiple testing using false discovery rate (FDR)^37^. Eicosanoids with FDR-adjusted *p* value < 0.05 were considered significant. Sex- and age-stratified analyses were also performed. We tested for effect modification by sex and age (< 65 vs. ≥ 65 years old) in relation to incident AF risk by including interaction terms in the Cox models. A joint score was also built to represent a weighted combination of top eicosanoids (FDR < 0.05). The score for sample *i* was defined as ${S}_{i}={\sum }_{j=1}^{n}{\beta }_{j}*{V}_{ij}$ , in which *n* is the number of top eicosanoids associated with incident AF, * β*_*j*_ is the estimate of effect size for eicosanoid *j*, and *V*_*ij*_ is the normalized level of eicosanoid *j* for sample *i*. Association of the joint score with incident AF was also tested using the Cox proportional hazards model and adjusted for clinical risk factors as the primary models.

In secondary analyses, we also examined the association of each eicosanoid with prevalent AF using generalized estimating equations. We adjusted for the same covariates as those used for the primary models. Eicosanoids with FDR < 0.05 were considered significant. All statistical analyses were performed using R software version 4.0.3 (https://www.r-project.org/).

## Results

### Study population

We included 2676 individuals without diagnosed AF (mean age 66 ± 9 years, 56% females) in the analysis of the association between eicosanoids and incident AF. Participants were followed for an average of 10.8 ± 3.4 years and a total of 351 participants were diagnosed with new-onset AF during the follow-up period. Baseline characteristics of the study population are presented in Table 1 and the baseline characteristics of subgroups are presented in sTable 1.

**Table 1:** Baseline characteristics of study sample.

Variable	Participants without prevalent AF n = 2676
Age (years)	66 ± 9
Women	1488 (55.6%)
Height (cm)	167 ± 10
Weight (kg)	79 ± 18
Current smoking	249 (9.3%)
Systolic blood pressure (mmHg)	129 ± 17
Diastolic blood pressure (mmHg)	74 ± 10
Antihypertensive medication use	1359 (50.8%)
Diabetes mellitus	342 (12.8%)
Prevalent heart failure	22 (0.8%)
Prevalent myocardial infarction	100 (3.7%)

Values are represented as n (%) for dichotomous variables or mean ± standard deviation (SD) for continuous variables.

### Association of eicosanoids with incident AF

As shown in Table 2, we identified six eicosanoids that were significantly associated with incident AF after adjusting for AF-related clinical risk factors^35^, including 9-oxoODE, EIC_33, 12(R) HETE, 9-oxoOTrE, 15 oxoEDE, and HETrE [M-H]. The full association results are depicted in sTable 2. For each eicosanoid, we provide the hazard ratio (HR) and 95% confidence interval (CI) per standard deviation of the eicosanoid. Increased concentrations of each of the six top eicosanoids were associated with increased risk of incident AF (HR ranging from 1.16 to 1.22). These associations remained significant after additional adjustment for inflammatory biomarkers CRP and IL-6. We further categorized each eicosanoid into tertiles, and displayed their association with incident AF through Kaplan–Meier plots (sFigs. 1–6).

**Table 2:** Eicosanoids associated with incident AF (FDR < 0.05).

Eicosanoids#	Putative identity	Primary model*			Exploratory model*		
		HR†	95% CI†	*P* value†	HR†	95% CI†	*P* value†
293.21136/5.1237	9-oxoODE	1.22	1.10–1.36	1.8 × 10^–4^	1.20	1.08–1.34	8.2 × 10^–4^
299.25921/5.5568	EIC_33	1.16	1.07–1.27	6.9 × 10^–4^	1.25	1.04–1.25	4.2 × 10^–3^
265.17938/3.7720	12(R) HETE	1.19	1.07–1.32	8.8 × 10^–4^	1.32	1.07–1.32	1.2 × 10^–3^
291.19445/4.3834	9-oxoOTrE	1.19	1.07–1.31	9.8 × 10^–4^	1.31	1.07–1.31	1.5 × 10^–3^
321.24360/6.1182	15 oxoEDE	1.18	1.07–1.29	1.0 × 10^–3^	1.28	1.05–1.28	4.0 × 10^–3^
321.24387/5.4933	HETrE [M-H]	1.17	1.06–1.29	1.6 × 10^–3^	1.27	1.04–1.27	5.8 × 10^–3^

^**#**^Each eicosanoid is represented by MZ/RT; MZ is the mass-to-charge ratio (to 5 decimals) and RT is the retention time (to 4 decimals).

*Primary model was adjusted for age, sex, height, weight, systolic and diastolic blood pressure, current smoking, use of antihypertensive medication, diabetes, history of myocardial infarction, and history of heart failure; Exploratory model was additionally adjusted for CRP and IL-6 concentration.

**†***HR* Hazard ratio expressed per standard deviation of log transformed normalized eicosanoid concentration, *CI* Confidence interval; *P* value was not adjusted for multiple testing.

**Figure 1:**
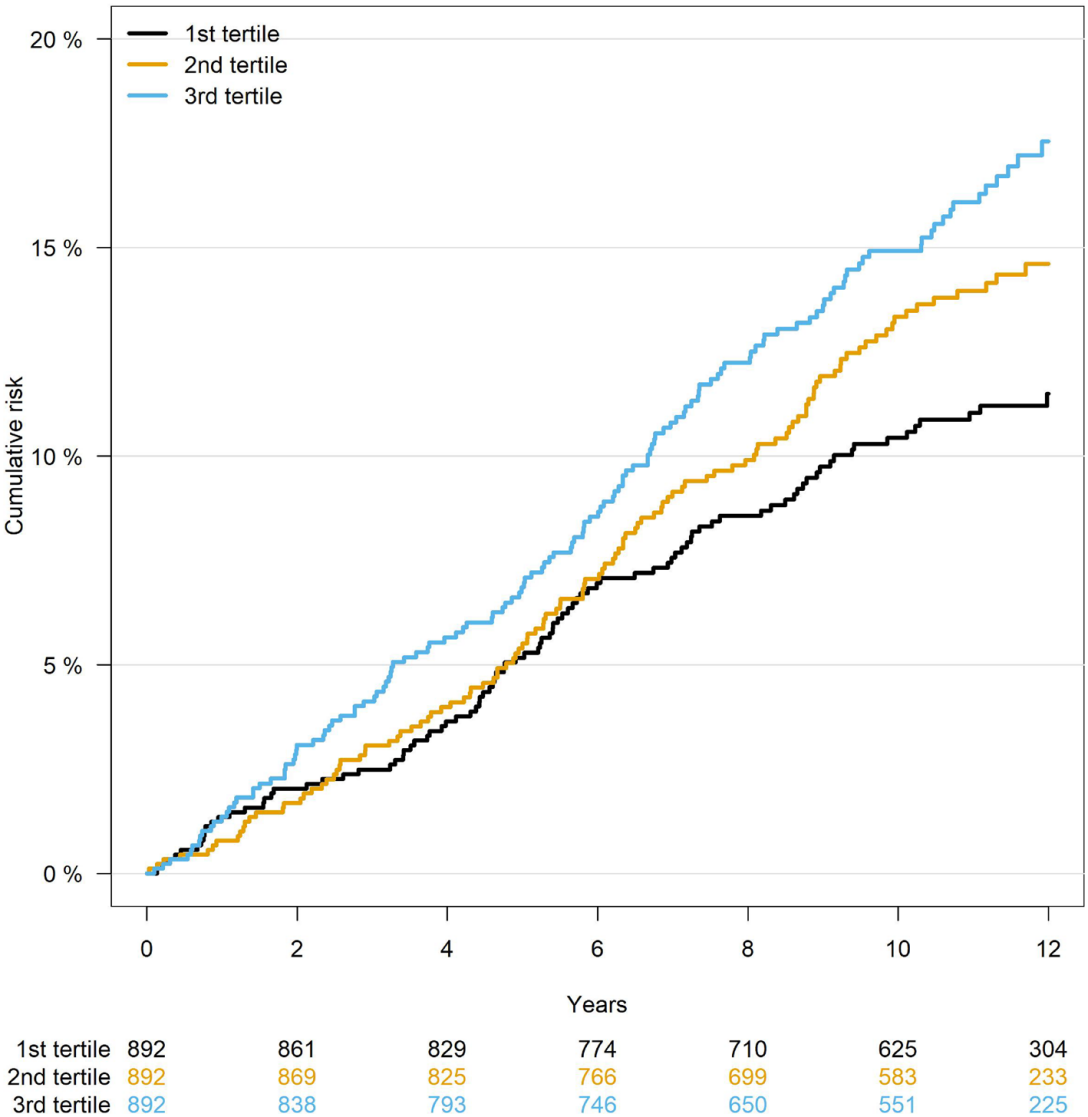
Cumulative risk curve of AF in tertile groups for the eicosanoid composite score. The score represented a weighted combination of six eicosanoids associated with incident AF. Participants were divided into three tertile groups based on their scores. Lower panel shows the number of participants at risk during the study period.

To assess the combined association of multiple eicosanoids with incident AF, we built a joint model that included the six top eicosanoids weighted by the effect size of individual eicosanoids, As displayed in Fig. 1, the score was significantly associated with incident AF after adjusting for known clinical risk factors (HR = 2.72, CI = 1.71–4.31, *P* = 2.1 × 10^–5^).

We also performed sex- and age-stratified analyses. As shown in sTable 3, only one eicosanoid (299.25921/5.5) was nominally significant for women, but all were significant in men. None of eicosanoids showed evidence of significant effect modification by sex. The associations of eicosanoids with incident AF also showed different patterns between older adults (≥ 65 years old) and younger people (< 65 years old) (sTable 4). Two eicosanoids, 293.21136/5.1237 and 321.24387/5.4933, showed relatively lower association in older adults compared to younger adults (*P value for interaction* = 0.02 and 0.01, respectively).

Finally, in sTable 5 we analyzed the association of each top eicosanoids with CRP and IL-6. We observed modest correlations, ranging from − 0.03 (9-oxoOTrE) to 0.13 (9-oxoODE) for CRP, and from − 0.4 (9-oxoOTrE) to 0.12 (9-oxoODE) for IL-6. Prevalent and incident AF cases tended to have higher levels of CRP and IL-6 compared with referents.

### Association of eicosanoids with prevalent AF

We also examined the association of eicosanoids with prevalent AF (n = 210). As depicted in sTable 2, five eicosanoids were significantly associated with prevalent AF after adjusting for AF-related clinical risk factors (FDR < 0.05)^35^. However, none of these were significantly associated with incident AF after accounting for multiple testing. Among the top six eicosanoids associated with incident AF, 9-oxoODE, EIC_33 and HETrE [M-H] were nominally associated with prevalent AF (*P* = 0.03, 0.02, and 0.009, respectively).

## Discussion

Eicosanoids are biologically active lipid mediators originated mostly from the omega-6 (n-6) PUFA, arachidonic acid, but also from substrates such as the (n−6) PUFA dihomo-γ-linolenic acid, and the omega-3 (n−3) PUFA eicosapentaenoic acid. Arachidonic acid, a key polyunsaturated fatty acid, is a precursor of many pro- and anti-inflammatory signaling molecules, including eicosanoids. Metabolomic profiling of arachidonic acid and its metabolites has improved our understanding of several cardiovascular diseases^24^. In the current study, we analyzed the association between eicosanoids and incident AF among FHS participants. We identified six eicosanoids—9-oxoODE, 12(R) HETE, 9-oxoOTrE, 15 oxoEDE, and HETrE, as well as an unknown eicosanoid^29^ (EIC_33)—that were significantly associated with incident AF after adjusting for known AF-related clinical risk factors. Three of these were nominally associated with prevalent AF.

### Eicosanoids associated with incident AF

Eicosanoid profiling in human plasma represents an opportunity to identify novel pathophysiologic biomarkers associated with AF initiation and progression. Relatively few investigations have assessed the distribution or abundance of plasma eicosanoids, and even fewer have sought to link this association with incident and prevalent AF.

A previous study described a directed non-targeted mass spectrometry approach for the discovery of eicosanoids and related oxylipins^29^. Members of the reported eicosanoid and related oxylipin metabolites described in this study were closely associated with markers of systemic inflammation. In our study, using similar approaches, we identified six eicosanoids that were significantly associated with incident AF. Interestingly, one of these eicosanoids (Mass-to-Charge Ratio/Retention Time: 293.21136/5.1237, Accurate Mass: 296.2351) has been identified as an epoxy fatty acid, main class: octadecanoids (LipidMaps ID: LMFA02000038; common name: 12,13 EpOME). For more details on this lipid, please see the link https://pubchem.ncbi.nlm.nih.gov/compound/5356421 and https://www.lipidmaps.org/data/LMSDRecord.php?LMID=LMFA02000038^29^. The 12,13-epoxyoctadecenoic acid (12,13-EpOME) is produced through the cytochrome P450 dependent metabolism and is known as isoleukotoxin^38^. The physiologic concentration of EpOME is reported to be dependent on the regulation of biosynthetic pathways and dietary intake of their relevant fatty acid, linoleic acid, which is the most abundantly consumed PUFA in the human diet^39^. In a study focused on the roles of linoleic acid metabolites in post-ischemic myocardial recovery, investigators showed that 12,13-EpOME and 12,13-DiHOME treated murine hearts exhibited reduced cardiac functional recovery after ischemia^40^.

### Oxo-octadecadienoic acid (9-OxoODE)

In a study on key regulatory processes promoting remarkable longevity in a representative Italian cohort, investigators have suggested that enhanced anti-oxidative response mechanisms might be activated. They observed decreased circulating levels of 9-oxoODE, a marker of lipid oxidative products of linoleic acid^41^. In contrast, obese individuals with nonalcoholic fatty liver disease have higher concentrations of plasma oxidized linoleic acid metabolites including 9- and 13-oxoODEs^42,43^. Obesity is a critical risk factor for AF, and a dietary study in obese youth showed that a 12 week treatment with a diet characterized by a low n6:n3 polyunsaturated fatty acids (PUFA) ratio decreased hepatic fat levels, including triglycerides and LDL, with evidence of improved insulin sensitivity. Oxidized linoleic acid metabolites including 9-oxoODE were reduced^44^. Atrial fatty infiltration contributes to abbreviation of action potential duration and a substrate for AF, and oxidized lipid metabolites likely contribute to this process^45^.

### Hydroxyeicosatetraenoic acids (HETEs)

HETEs are metabolites of arachidonic acid produced in lipoxygenase pathway. Arachidonic acid is involved in several physiological and pathophysiological processes including development of cardiovascular diseases^46^, hypertension^47^, cardiac hypertrophy^47,48^, and inflammatory disease conditions^49,50^. 12-HETE has been reported to act as a vasoconstrictor and implicated in heart failure by induction of cardiac fibrosis^51^. Increased levels of the eicosanoid 12-HETE have been observed in the serum of patients with newly-diagnosed Type 1 diabetes^52^ and heart failure with preserved ejection fraction in patients with type 2 diabetes^53^. Huang et al.^54^ observed that the incidence of future acute myocardial infarction was more frequently reported in patients with higher baseline levels of numerous HETEs including the 12-HETE when compared to their counterparts. Finally, there is an evidence of pro-inflammatory property of HETEs^55^. It has been reported that, the levels of 12-HETE and 5-HETE were significantly increased in individuals with low-grade inflammation and obesity. Importantly, the levels of 12-HETE, 5-HETE, and TNF-α significantly decreased after weight reduction^55^.

### Isolevuglandins in hypertension and AF

In the setting of increased oxidative stress, arachidonic acid and its metabolites are readily oxidized by free radicals, leading to formation of highly reactive electrophiles (isolevuglandins) that can form adducts with proteins with which they interact and modify their function^56^. These protein adducts are reported to have increased abundance in plasma from patients with atherosclerosis and end-stage renal disease^56^ as well as hypertension and AF^57–59^. Quantitation of isolevuglandins requires a different protocol than the approach used to quantify eicosanoids in the current study^57^. A clinical trial is underway to test the hypothesis that isolevuglandin scavengers can prevent the recurrence of AF following catheter ablation (NCT04433091)^60^.

Altogether, the results of our study as well as the previous reports may support the notion that specific eicosanoids may serve as biomarkers for prediction of AF incidence. Studies that clarify the role of these lipids in AF may provide novel insights into the aging-related role of these lipids, and guide the selective targeting of patients for whom scavenging these compounds may be helpful.

### Clinical implications and future directions

Our study reported the detection of eicosanoids in human plasma represent biomarkers predictive for incident AF. Detected eicosanoids could initially exist in the plasma due to systemic inflammatory response associated with pathologies and risk factors such as cardiovascular diseases, diabetes, aging, etc., that may predispose to incident AF. However, it may also be possible that eicosanoids are generated within the diseased atrial tissues, and therefore, persistent inflammatory mediators may be released and detected in the plasma. Other eicosanoids and metabolites below measurable range remain to be metabolized and therefore need to be localized and measured in the diseased atrial tissues. Therefore, comparative studies of eicosanoid levels in individual-based paired-samples in plasma versus atrial tissues from healthy individuals and AF patients at various stages of disease could provide valuable insights into these metabolites' potential contributions to the initiation and progression of AF.

Finally, in conjunction with the advancement of recent approaches, profiling studies may facilitate integration of clinical information with multiomics data^61^. Combining these data with genomic^62,63^, transcriptomic^64^, proteomic^65,66^, and metabolomics^67^ datasets may provide deeper insights into the underlying mechanisms of AF, from initiation to recurrence to progression, and expedite the design of targeted intervention and therapeutic strategies. Future studies may aim to characterize the eicosanoid metabolites and identify their precise involvement in signaling pathways, i.e., their production, degradation, and activities that may lead to pro-, anti-, or resolving-inflammatory processes more deeply. Such efforts are critical in advancing our understanding the role of eicosanoids mediating AF initiation and progression.

## Limitations

We acknowledge several epidemiological, clinical, laboratory, and statistical limitations. First, the vast majority of the study participants were of European ancestry and the mean age was 66 years; therefore the generalizability of the results to other races/ethnicities and younger individuals is unknown. Secondly, silent, paroxysmal AF was very likely unrecognized, leading to some misclassification of prevalent and incident AF. Also, we had limited information about AF type (paroxysmal, persistent, longstanding-persistent). In addition, although we describe associations, we cannot determine causal relations or rule out residual confounding. Laboratory limitations include possible measurement errors in biomarker profiles. Statistical limitations include that subgroup analyses (men vs. women and younger (< 65 years) vs. older (≥ 65 years) may be affected by lack of statistical power. Given the short half-life of eicosanoids, evaluation of the abundance of more stable (and pro-inflammatory) metabolites (e.g., 1,2-diols, and dihydroxyeicosatrienoic acids) may be easier to detect than the anti-inflammatory eicosanoids. Finally, replication of our results in other trial cohorts is needed.

## Conclusions

In our study, six eicosanoids were significantly associated with incident AF after adjust for multiple testing, of which three were nominally associated with prevalent AF. Eicosanoids are oxidized lipid metabolites that are associated with impaired atrial insulin sensitivity and changes in atrial structure and electrophysiology. Thus, eicosanoid metabolomics profiling may enhance our understanding of the abundance of risk-associated eicosanoid metabolites and facilitate the development of inhibitors of eicosanoid synthesis and activity. A deeper understanding of eicosanoids in relation to AF may have significant clinical implications for AF prevention and therapeutic development. Future studies are needed to replicate and examine the biological bases of our findings.

## Supplementary Information

Supplementary Information.

## Data Availability

The authors confirm that the data supporting the findings of this study are available within the article and its supplementary materials.
