# Supplementary material for: The association between eicosanoids and incident atrial fibrillation in the Framingham Heart Study
Source: Sci Rep. 2022 Nov 23;12:20218. doi: 10.1038/s41598-022-21786-0 (PMC9684401; doi:10.1038/s41598-022-21786-0)

**The association between eicosanoids and incident atrial fibrillation in the Framingham Heart Study – Supplementary Material**

**Table of Contents**

**sTable 1**. Baseline characteristics of study sample in subgroups **2**

**sTable 2**. Association of eicosanoids with incident AF and prevalent AF **3**

**sTable 3**. Top AF-related eicosanoids with sex-stratified analysis **8**

**sTable 4**. Top AF-related eicosanoids with age-stratified analysis **9**

**sTable 5**. Correlation analysis with CRP and IL-6 **10**

**sFigure 1**. Survival curves for 293.21136/5.1237 **11**

**sFigure 2**. Survival curves for 299.25921/5.5568 **12**

**sFigure 3**. Survival curves for 265.17938/3.7720 **13**

**sFigure 4**. Survival curves for 291.19445/4.3834 **14**

**sFigure 5**. Survival curves for 321.24360/6.1182 **15**

**sFigure 6**. Survival curves for 321.24387/5.4933 **16**

**sTable 1**. Baseline characteristics of study sample in subgroups

| **Variable** | **Prevalent AF**  **(n=210)** | **Incident AF**  **(n=351)** | **AF-free**  **(n=2325)** |
| --- | --- | --- | --- |
| Age (years) | 73 ± 9 | 71 ± 8 | 66 ± 9 |
| Women | 77 (36.7%) | 151 (43.0%) | 1337 (57.5%) |
| Height (cm) | 169 ± 10 | 168 ± 10 | 167 ± 9 |
| Weight (kg) | 85 ± 21 | 82 ± 19 | 78 ± 17 |
| Current smoking | 9 (4.3%) | 24 (6.8%) | 225 (9.7%) |
| Systolic blood pressure (mmHg) | 128 ± 20 | 134 ± 18 | 128 ± 17 |
| Diastolic blood pressure (mmHg) | 70 ± 10 | 72 ± 11 | 74 ± 10 |
| Antihypertensive medication use | 192 (91.4%) | 230 (65.5%) | 1129 (48.6%) |
| Diabetes mellitus | 55 (26.2%) | 71 (20.2%) | 271 (11.7%) |
| Prevalent heart failure | 33 (15.7%) | 5 (1.4%) | 17 (0.7%) |
| Prevalent myocardial infarction | 52 (24.8%) | 23 (6.6%) | 77 (3.3%) |
| CRP, median (25%, 75%) | 2.2 (1.1, 4.2) | 1.7 (0.9, 3.3) | 1.4 (0.7, 3.1) |
| IL-6, median (25%, 75%) | 2.6 (1.6, 4.9) | 2.2 (1.5, 3.3) | 1.7 (1.1, 2.7) |

Values are represented as n (%) for dichotomous variables, or mean ± Standard Deviation (SD) for continuous variables.

**sTable 2**. Association of eicosanoids with incident and prevalent AF

| **Eicosanoids**^#^ | **Incident AF** | | | | **Prevalent AF** | | | |
| --- | --- | --- | --- | --- | --- | --- | --- | --- |
|  | HR^†^ | 95% CI**^†^** | P-value**^†^** | FDR | OR^†^ | 95% CI**^†^** | P-value**^†^** | FDR |
| **293.21136/5.1237** | 1.22 | 1.10-1.36 | 1.8E-04 | 0.03 | 1.19 | 1.02-1.39 | 2.7E-02 | 0.27 |
| **299.25921/5.5568** | 1.16 | 1.07-1.27 | 6.9E-04 | 0.03 | 1.23 | 1.03-1.46 | 2.2E-02 | 0.26 |
| **265.17938/3.7720** | 1.19 | 1.07-1.32 | 8.8E-04 | 0.03 | 1.05 | 0.89-1.23 | 5.7E-01 | 0.82 |
| **291.19445/4.3834** | 1.19 | 1.07-1.31 | 9.8E-04 | 0.03 | 1.10 | 0.94-1.29 | 2.5E-01 | 0.63 |
| **321.24360/6.1182** | 1.18 | 1.07-1.29 | 1.0E-03 | 0.03 | 1.12 | 0.96-1.32 | 1.5E-01 | 0.59 |
| **321.24387/5.4933** | 1.17 | 1.06-1.29 | 1.6E-03 | 0.04 | 1.24 | 1.05-1.46 | 9.3E-03 | 0.17 |
| **295.22746/5.6331** | 1.16 | 1.05-1.29 | 3.6E-03 | 0.08 | 1.03 | 0.88-1.20 | 7.5E-01 | 0.90 |
| **319.22993/5.7615** | 1.15 | 1.04-1.27 | 6.5E-03 | 0.13 | 1.10 | 0.94-1.29 | 2.3E-01 | 0.61 |
| **331.26376/6.2747** | 0.83 | 0.73-0.95 | 7.2E-03 | 0.13 | 0.89 | 0.78-1.01 | 6.2E-02 | 0.46 |
| **295.22748/4.1506** | 1.14 | 1.03-1.25 | 8.5E-03 | 0.14 | 1.12 | 0.96-1.31 | 1.6E-01 | 0.59 |
| **321.23876/5.1784** | 1.12 | 1.03-1.23 | 9.6E-03 | 0.14 | 1.21 | 1.01-1.44 | 3.6E-02 | 0.30 |
| **295.22767/4.5456** | 1.13 | 1.03-1.25 | 1.1E-02 | 0.14 | 1.29 | 1.09-1.52 | 2.3E-03 | 6.8E-02 |
| **337.23690/3.16** | 0.91 | 0.84-0.98 | 1.2E-02 | 0.15 | 0.90 | 0.71-1.15 | 4.1E-01 | 0.73 |
| **293.20771/3.8806** | 1.13 | 1.02-1.24 | 1.5E-02 | 0.17 | 1.13 | 0.96-1.33 | 1.4E-01 | 0.56 |
| **379.24670/5.8936** | 0.87 | 0.78-0.98 | 1.8E-02 | 0.18 | 0.94 | 0.81-1.09 | 3.9E-01 | 0.73 |
| **345.24354/4.9537** | 1.11 | 1.02-1.22 | 1.8E-02 | 0.18 | 1.10 | 0.89-1.36 | 3.7E-01 | 0.72 |
| **395.24120/4.5146** | 1.11 | 1.02-1.21 | 1.9E-02 | 0.18 | 1.10 | 0.91-1.34 | 3.3E-01 | 0.69 |
| **345.24356/5.610** | 1.11 | 1.02-1.22 | 2.2E-02 | 0.20 | 1.12 | 0.92-1.36 | 2.6E-01 | 0.63 |
| **265.17954/3.8883** | 1.09 | 1.01-1.17 | 2.3E-02 | 0.20 | 0.96 | 0.72-1.28 | 7.8E-01 | 0.92 |
| **317.21464/4.9915** | 1.13 | 1.01-1.25 | 2.6E-02 | 0.21 | 1.04 | 0.89-1.22 | 6.0E-01 | 0.84 |
| **353.23290/4.5146** | 1.13 | 1.01-1.26 | 2.9E-02 | 0.22 | 1.20 | 1.03-1.40 | 1.9E-02 | 0.25 |
| **297.24340/4.7785** | 1.12 | 1.01-1.25 | 2.9E-02 | 0.22 | 1.18 | 1.01-1.37 | 3.5E-02 | 0.30 |
| **293.21060/4.8458** | 1.11 | 1.01-1.22 | 3.9E-02 | 0.27 | 1.05 | 0.89-1.23 | 5.7E-01 | 0.82 |
| **265.17779/3.6499** | 1.11 | 1.00-1.24 | 5.1E-02 | 0.33 | 1.00 | 0.85-1.17 | 9.6E-01 | 0.99 |
| **317.21465/5.3600** | 1.11 | 1.00-1.23 | 5.2E-02 | 0.33 | 1.11 | 0.95-1.30 | 1.9E-01 | 0.60 |
| **319.22690/5.1316** | 1.08 | 1.00-1.18 | 5.4E-02 | 0.33 | 1.24 | 1.03-1.49 | 2.3E-02 | 0.26 |
| **297.24327/5.532** | 1.11 | 1.00-1.22 | 5.5E-02 | 0.33 | 1.11 | 0.95-1.29 | 2.0E-01 | 0.60 |
| **279.19630/3.6422** | 0.94 | 0.88-1.01 | 7.5E-02 | 0.43 | 1.63 | 1.24-2.14 | 5.2E-04 | 2.0E-02 |
| **321.23900/6.2592** | 1.09 | 0.99-1.20 | 9.0E-02 | 0.49 | 1.25 | 1.05-1.48 | 1.1E-02 | 0.18 |
| **293.21077/5.5421** | 1.09 | 0.99-1.21 | 9.1E-02 | 0.49 | 1.23 | 1.05-1.44 | 8.5E-03 | 0.16 |
| **265.17710/3.4855** | 1.08 | 0.98-1.20 | 1.0E-01 | 0.53 | 0.90 | 0.75-1.09 | 2.8E-01 | 0.64 |
| **295.22813/4.9691** | 1.09 | 0.98-1.20 | 1.1E-01 | 0.53 | 1.18 | 1.00-1.39 | 4.4E-02 | 0.36 |
| **353.23340/2.758** | 1.07 | 0.99-1.15 | 1.1E-01 | 0.53 | 1.16 | 0.92-1.47 | 2.1E-01 | 0.61 |
| **327.21621/3.5010** | 0.92 | 0.84-1.02 | 1.2E-01 | 0.54 | 1.01 | 0.86-1.18 | 9.4E-01 | 0.99 |
| **343.22647/4.8458** | 1.08 | 0.98-1.19 | 1.2E-01 | 0.54 | 1.10 | 0.93-1.30 | 2.8E-01 | 0.64 |
| **375.21752/4.8458** | 1.06 | 0.98-1.15 | 1.3E-01 | 0.54 | 0.95 | 0.75-1.19 | 6.5E-01 | 0.87 |
| **381.26055/5.8389** | 1.08 | 0.98-1.20 | 1.3E-01 | 0.54 | 1.24 | 1.06-1.44 | 7.0E-03 | 0.15 |
| **317.21133/4.4230** | 1.07 | 0.98-1.17 | 1.3E-01 | 0.54 | 1.15 | 0.96-1.37 | 1.4E-01 | 0.57 |
| **303.23285/4.5301** | 1.04 | 0.99-1.10 | 1.3E-01 | 0.54 | 1.14 | 0.80-1.61 | 4.7E-01 | 0.79 |
| **291.19731/4.7564** | 1.08 | 0.98-1.20 | 1.3E-01 | 0.54 | 1.13 | 0.97-1.33 | 1.2E-01 | 0.55 |
| **327.21704/2.7054** | 0.93 | 0.84-1.02 | 1.4E-01 | 0.54 | 0.93 | 0.79-1.10 | 4.1E-01 | 0.74 |
| **379.24881/4.7785** | 1.08 | 0.97-1.20 | 1.4E-01 | 0.54 | 0.97 | 0.83-1.14 | 7.4E-01 | 0.89 |
| **289.18052/4.1822** | 1.05 | 0.98-1.11 | 1.4E-01 | 0.54 | 1.10 | 0.84-1.43 | 5.0E-01 | 0.79 |
| **327.21730/4.7564** | 0.96 | 0.90-1.02 | 1.5E-01 | 0.54 | 1.08 | 0.82-1.41 | 5.9E-01 | 0.83 |
| **327.21967/2.9775** | 0.94 | 0.87-1.03 | 1.7E-01 | 0.59 | 0.96 | 0.79-1.17 | 7.1E-01 | 0.89 |
| **353.23279/2.4213** | 1.07 | 0.97-1.18 | 1.7E-01 | 0.59 | 1.25 | 1.06-1.48 | 9.7E-03 | 0.17 |
| **325.20067/2.6667** | 0.96 | 0.92-1.02 | 1.8E-01 | 0.59 | 0.79 | 0.57-1.08 | 1.4E-01 | 0.56 |
| **341.23417/3.7566** | 0.94 | 0.85-1.03 | 1.8E-01 | 0.59 | 0.89 | 0.76-1.05 | 1.8E-01 | 0.59 |
| **315.25451/4.1352** | 0.94 | 0.85-1.03 | 1.8E-01 | 0.59 | 1.01 | 0.85-1.19 | 9.2E-01 | 0.98 |
| **343.22611/4.9615** | 1.07 | 0.97-1.17 | 1.8E-01 | 0.59 | 1.18 | 0.99-1.41 | 6.0E-02 | 0.45 |
| **381.26094/5.5568** | 1.07 | 0.97-1.19 | 1.9E-01 | 0.59 | 1.20 | 1.02-1.40 | 2.7E-02 | 0.27 |
| **343.22624/4.3058** | 1.07 | 0.97-1.18 | 2.0E-01 | 0.60 | 1.16 | 0.98-1.36 | 8.3E-02 | 0.48 |
| **331.18905/3.6654** | 1.06 | 0.97-1.17 | 2.0E-01 | 0.60 | 1.16 | 0.99-1.37 | 7.3E-02 | 0.47 |
| **329.23268/2.8760** | 0.96 | 0.89-1.03 | 2.1E-01 | 0.62 | 0.84 | 0.67-1.06 | 1.5E-01 | 0.59 |
| **313.23862/5.2172** | 1.06 | 0.96-1.17 | 2.2E-01 | 0.63 | 1.13 | 0.96-1.33 | 1.3E-01 | 0.56 |
| **341.23302/3.6189** | 0.94 | 0.85-1.04 | 2.2E-01 | 0.63 | 1.01 | 0.86-1.19 | 8.7E-01 | 0.96 |
| **299.25274/4.5146** | 1.06 | 0.96-1.16 | 2.3E-01 | 0.64 | 1.01 | 0.85-1.20 | 9.1E-01 | 0.97 |
| **353.23231/2.3281** | 0.95 | 0.87-1.04 | 2.6E-01 | 0.72 | 0.80 | 0.67-0.95 | 1.3E-02 | 0.20 |
| **291.19448/4.578** | 1.06 | 0.96-1.16 | 2.6E-01 | 0.72 | 0.92 | 0.78-1.10 | 3.7E-01 | 0.72 |
| **335.22381/5.924** | 1.06 | 0.96-1.17 | 2.7E-01 | 0.73 | 1.15 | 0.98-1.35 | 9.7E-02 | 0.51 |
| **343.22600/4.6227** | 1.04 | 0.97-1.11 | 2.8E-01 | 0.74 | 1.09 | 0.84-1.42 | 5.2E-01 | 0.80 |
| **355.24341/2.1231** | 1.05 | 0.96-1.14 | 2.9E-01 | 0.75 | 1.19 | 0.96-1.46 | 1.1E-01 | 0.53 |
| **353.22885/4.7711** | 1.05 | 0.95-1.15 | 3.3E-01 | 0.83 | 1.30 | 1.09-1.56 | 4.0E-03 | 0.10 |
| **375.22037/2.3825** | 1.04 | 0.95-1.14 | 3.5E-01 | 0.83 | 0.79 | 0.65-0.95 | 1.3E-02 | 0.20 |
| **329.23350/2.5064** | 0.99 | 0.95-1.02 | 3.5E-01 | 0.83 | 0.69 | 0.41-1.16 | 1.6E-01 | 0.59 |
| **335.22212/2.8533** | 0.97 | 0.90-1.04 | 3.5E-01 | 0.83 | 0.83 | 0.66-1.05 | 1.3E-01 | 0.56 |
| **355.24341/2.5442** | 0.98 | 0.94-1.02 | 3.6E-01 | 0.83 | 0.32 | 0.22-0.45 | 1.6E-10 | 1.3E-08 |
| **321.24000/6.3408** | 1.05 | 0.95-1.16 | 3.6E-01 | 0.83 | 1.11 | 0.94-1.31 | 2.2E-01 | 0.61 |
| **341.21075/5.5039** | 1.05 | 0.94-1.17 | 3.7E-01 | 0.83 | 1.08 | 0.93-1.26 | 3.2E-01 | 0.69 |
| **329.23303/2.6750** | 0.98 | 0.92-1.03 | 3.7E-01 | 0.83 | 0.84 | 0.62-1.14 | 2.6E-01 | 0.63 |
| **331.18981/2.6978** | 0.97 | 0.90-1.04 | 3.7E-01 | 0.83 | 0.99 | 0.80-1.21 | 8.8E-01 | 0.96 |
| **395.23911/3.4235** | 1.04 | 0.95-1.13 | 3.7E-01 | 0.83 | 0.94 | 0.77-1.15 | 5.3E-01 | 0.80 |
| **315.19534/2.7282** | 1.03 | 0.96-1.11 | 3.8E-01 | 0.83 | 0.81 | 0.64-1.03 | 8.5E-02 | 0.48 |
| **359.22047/5.689** | 0.95 | 0.84-1.07 | 3.8E-01 | 0.84 | 1.00 | 0.87-1.14 | 9.7E-01 | 0.99 |
| **299.25251/4.4540** | 0.95 | 0.86-1.06 | 4.0E-01 | 0.85 | 1.10 | 0.94-1.27 | 2.4E-01 | 0.61 |
| **293.20906/4.6304** | 1.04 | 0.95-1.14 | 4.2E-01 | 0.88 | 1.09 | 0.92-1.29 | 3.1E-01 | 0.67 |
| **339.26366/3.4389** | 0.96 | 0.86-1.06 | 4.2E-01 | 0.88 | 0.93 | 0.80-1.08 | 3.6E-01 | 0.72 |
| **395.24134/2.6439** | 1.03 | 0.96-1.10 | 4.4E-01 | 0.90 | 0.99 | 0.76-1.28 | 9.1E-01 | 0.97 |
| **357.20394/5.298** | 1.03 | 0.95-1.11 | 4.4E-01 | 0.90 | 0.99 | 0.80-1.24 | 9.4E-01 | 0.99 |
| **317.26215/3.3995** | 0.97 | 0.88-1.06 | 4.5E-01 | 0.91 | 1.00 | 0.84-1.20 | 9.8E-01 | 1.00 |
| **343.22842/4.7564** | 1.04 | 0.94-1.15 | 4.7E-01 | 0.94 | 1.16 | 0.98-1.36 | 8.1E-02 | 0.48 |
| **335.22534/5.2172** | 1.04 | 0.93-1.15 | 5.0E-01 | 0.96 | 1.14 | 0.97-1.33 | 1.1E-01 | 0.52 |
| **315.19812/4.1045** | 1.04 | 0.93-1.16 | 5.2E-01 | 0.96 | 1.07 | 0.93-1.23 | 3.7E-01 | 0.72 |
| **353.23472/3.7643** | 0.97 | 0.87-1.07 | 5.2E-01 | 0.96 | 1.04 | 0.89-1.21 | 6.5E-01 | 0.87 |
| **319.22279/3.9504** | 0.97 | 0.87-1.08 | 5.5E-01 | 0.96 | 1.07 | 0.91-1.25 | 4.1E-01 | 0.74 |
| **379.24802/5.532** | 1.03 | 0.93-1.14 | 5.5E-01 | 0.96 | 1.07 | 0.91-1.26 | 4.0E-01 | 0.73 |
| **325.19843/2.2013** | 0.98 | 0.91-1.05 | 5.5E-01 | 0.96 | 0.33 | 0.26-0.42 | 1.4E-20 | 2.0E-18 |
| **330.29721/5.4145** | 0.97 | 0.87-1.08 | 5.6E-01 | 0.96 | 1.10 | 0.94-1.28 | 2.5E-01 | 0.63 |
| **309.20502/2.8092** | 1.02 | 0.95-1.10 | 5.6E-01 | 0.96 | 0.82 | 0.65-1.03 | 8.5E-02 | 0.48 |
| **321.24005/4.6227** | 0.97 | 0.88-1.07 | 5.7E-01 | 0.96 | 1.03 | 0.86-1.22 | 7.7E-01 | 0.92 |
| **317.20817/3.9734** | 1.03 | 0.92-1.15 | 5.8E-01 | 0.96 | 1.05 | 0.91-1.22 | 4.9E-01 | 0.79 |
| **327.27826/5.845** | 1.03 | 0.92-1.15 | 5.9E-01 | 0.96 | 1.03 | 0.89-1.18 | 7.2E-01 | 0.89 |
| **311.22334/4.4914** | 1.03 | 0.93-1.13 | 5.9E-01 | 0.96 | 1.16 | 0.98-1.36 | 7.9E-02 | 0.48 |
| **315.25406/3.9190** | 0.97 | 0.88-1.07 | 5.9E-01 | 0.96 | 0.99 | 0.83-1.17 | 8.7E-01 | 0.96 |
| **327.21636/2.8607** | 0.98 | 0.89-1.07 | 5.9E-01 | 0.96 | 0.82 | 0.69-0.97 | 2.2E-02 | 0.26 |
| **313.23708/3.4235** | 0.97 | 0.87-1.08 | 6.0E-01 | 0.96 | 1.08 | 0.92-1.25 | 3.4E-01 | 0.71 |
| **303.23198/4.8920** | 0.99 | 0.93-1.04 | 6.0E-01 | 0.96 | 0.94 | 0.71-1.24 | 6.5E-01 | 0.87 |
| **327.27832/4.5610** | 0.97 | 0.88-1.08 | 6.1E-01 | 0.96 | 1.11 | 0.95-1.29 | 1.8E-01 | 0.59 |
| **333.20420/4.4840** | 1.03 | 0.93-1.14 | 6.1E-01 | 0.96 | 1.19 | 1.01-1.39 | 3.6E-02 | 0.30 |
| **337.23499/3.6267** | 1.03 | 0.93-1.13 | 6.2E-01 | 0.96 | 1.11 | 0.94-1.30 | 2.2E-01 | 0.61 |
| **315.19275/3.7049** | 0.97 | 0.86-1.10 | 6.2E-01 | 0.96 | 0.98 | 0.86-1.12 | 7.3E-01 | 0.89 |
| **330.29699/5.1699** | 0.97 | 0.87-1.09 | 6.2E-01 | 0.96 | 1.08 | 0.93-1.25 | 3.0E-01 | 0.67 |
| **287.16743/3.6739** | 0.98 | 0.93-1.05 | 6.2E-01 | 0.96 | 0.95 | 0.73-1.24 | 7.1E-01 | 0.89 |
| **317.21030/3.8652** | 1.03 | 0.93-1.14 | 6.3E-01 | 0.96 | 1.04 | 0.89-1.22 | 5.9E-01 | 0.83 |
| **335.22072/3.9657** | 1.02 | 0.93-1.12 | 6.4E-01 | 0.96 | 1.08 | 0.92-1.28 | 3.6E-01 | 0.72 |
| **303.23071/5.1159** | 0.98 | 0.91-1.06 | 6.4E-01 | 0.96 | 0.98 | 0.80-1.20 | 8.4E-01 | 0.95 |
| **335.22033/2.6439** | 1.02 | 0.94-1.10 | 6.5E-01 | 0.96 | 0.96 | 0.77-1.19 | 6.9E-01 | 0.89 |
| **353.23357/4.2834** | 1.02 | 0.92-1.14 | 6.5E-01 | 0.96 | 1.14 | 0.98-1.32 | 8.8E-02 | 0.48 |
| **349.20164/2.6750** | 1.02 | 0.92-1.14 | 6.5E-01 | 0.96 | 1.01 | 0.86-1.20 | 8.8E-01 | 0.96 |
| **373.25260/1.8727** | 1.02 | 0.92-1.13 | 6.8E-01 | 0.98 | 1.06 | 0.90-1.26 | 4.6E-01 | 0.78 |
| **309.20669/3.9274** | 0.98 | 0.89-1.08 | 6.9E-01 | 0.98 | 1.01 | 0.86-1.18 | 9.3E-01 | 0.98 |
| **351.21350/2.5519** | 0.99 | 0.95-1.03 | 7.0E-01 | 0.98 | 0.07 | 0.04-0.10 | 4.2E-39 | 2.3E-36 |
| **315.19347/3.8337** | 0.98 | 0.87-1.10 | 7.0E-01 | 0.98 | 1.02 | 0.89-1.18 | 7.4E-01 | 0.89 |
| **345.24247/5.4146** | 0.99 | 0.93-1.05 | 7.2E-01 | 0.98 | 1.06 | 0.80-1.40 | 7.0E-01 | 0.89 |
| **315.19577/2.9853** | 0.98 | 0.87-1.10 | 7.2E-01 | 0.98 | 1.03 | 0.90-1.18 | 6.9E-01 | 0.89 |
| **361.23672/3.5879** | 0.98 | 0.90-1.08 | 7.4E-01 | 0.98 | 0.94 | 0.80-1.11 | 4.7E-01 | 0.79 |
| **367.20971/1.6804** | 0.98 | 0.88-1.10 | 7.5E-01 | 0.98 | 1.01 | 0.88-1.17 | 8.4E-01 | 0.95 |
| **315.19295/4.4067** | 1.02 | 0.91-1.13 | 7.5E-01 | 0.98 | 0.99 | 0.85-1.14 | 8.5E-01 | 0.96 |
| **317.21175/4.1899** | 1.02 | 0.92-1.13 | 7.6E-01 | 0.98 | 1.15 | 0.98-1.34 | 8.6E-02 | 0.48 |
| **335.22219/3.959** | 1.01 | 0.93-1.10 | 7.7E-01 | 0.98 | 1.03 | 0.86-1.24 | 7.4E-01 | 0.90 |
| **317.21255/3.4544** | 0.99 | 0.88-1.10 | 7.9E-01 | 0.98 | 1.00 | 0.86-1.16 | 9.5E-01 | 0.99 |
| **361.24043/4.1276** | 0.99 | 0.88-1.10 | 7.9E-01 | 0.98 | 1.00 | 0.86-1.15 | 9.8E-01 | 1.00 |
| **325.26572/4.1275** | 1.01 | 0.91-1.13 | 8.0E-01 | 0.98 | 1.08 | 0.92-1.25 | 3.5E-01 | 0.71 |
| **353.23443/4.1506** | 0.99 | 0.88-1.10 | 8.0E-01 | 0.98 | 1.06 | 0.91-1.23 | 4.4E-01 | 0.77 |
| **313.23868/5.376** | 0.99 | 0.89-1.09 | 8.0E-01 | 0.98 | 1.11 | 0.94-1.30 | 2.3E-01 | 0.61 |
| **357.25889/1.8650** | 0.99 | 0.90-1.09 | 8.1E-01 | 0.98 | 0.97 | 0.82-1.14 | 7.1E-01 | 0.89 |
| **367.20771/2.5064** | 0.99 | 0.90-1.08 | 8.1E-01 | 0.98 | 0.97 | 0.82-1.16 | 7.6E-01 | 0.91 |
| **355.24324/2.2159** | 1.01 | 0.96-1.05 | 8.1E-01 | 0.98 | 1.25 | 0.88-1.77 | 2.1E-01 | 0.61 |
| **395.24290/3.5406** | 1.01 | 0.92-1.12 | 8.2E-01 | 0.98 | 0.96 | 0.80-1.14 | 6.2E-01 | 0.84 |
| **355.25140/4.3989** | 0.99 | 0.90-1.09 | 8.4E-01 | 0.98 | 0.97 | 0.83-1.14 | 7.2E-01 | 0.89 |
| **349.20230/1.1295** | 1.01 | 0.89-1.16 | 8.4E-01 | 0.98 | 0.95 | 0.84-1.07 | 3.9E-01 | 0.73 |
| **323.22167/4.4230** | 0.99 | 0.90-1.09 | 8.5E-01 | 0.98 | 1.11 | 0.95-1.31 | 1.9E-01 | 0.60 |
| **335.22411/4.4463** | 1.01 | 0.91-1.11 | 8.5E-01 | 0.98 | 1.11 | 0.94-1.30 | 2.2E-01 | 0.61 |
| **367.21565/3.3064** | 1.01 | 0.92-1.11 | 8.6E-01 | 0.98 | 1.11 | 0.93-1.32 | 2.4E-01 | 0.61 |
| **373.25363/1.4951** | 0.99 | 0.87-1.13 | 8.6E-01 | 0.98 | 0.91 | 0.81-1.04 | 1.6E-01 | 0.59 |
| **317.20859/4.5533** | 0.99 | 0.89-1.10 | 8.6E-01 | 0.98 | 1.08 | 0.93-1.25 | 3.3E-01 | 0.70 |
| **389.19426/4.655** | 1.01 | 0.94-1.07 | 8.6E-01 | 0.98 | 1.09 | 0.84-1.41 | 5.2E-01 | 0.80 |
| **357.25889/2.4129** | 1.01 | 0.91-1.12 | 8.7E-01 | 0.98 | 1.04 | 0.88-1.22 | 6.6E-01 | 0.87 |
| **395.24057/2.8760** | 1.01 | 0.94-1.07 | 8.7E-01 | 0.98 | 1.10 | 0.85-1.42 | 4.7E-01 | 0.79 |
| **287.16625/3.7643** | 0.99 | 0.82-1.18 | 8.8E-01 | 0.98 | 0.98 | 0.90-1.08 | 7.0E-01 | 0.89 |
| **291.19595/4.8689** | 0.99 | 0.90-1.10 | 8.8E-01 | 0.98 | 1.13 | 0.96-1.33 | 1.3E-01 | 0.56 |
| **323.21961/4.119** | 1.01 | 0.91-1.11 | 8.8E-01 | 0.98 | 1.09 | 0.93-1.28 | 3.1E-01 | 0.67 |
| **345.24062/4.3912** | 1.01 | 0.91-1.12 | 8.9E-01 | 0.98 | 0.89 | 0.76-1.05 | 1.7E-01 | 0.59 |
| **351.21430/3.8806** | 0.99 | 0.89-1.11 | 9.0E-01 | 0.98 | 1.02 | 0.88-1.19 | 8.0E-01 | 0.94 |
| **343.22663/4.4617** | 1.00 | 0.93-1.06 | 9.0E-01 | 0.98 | 1.15 | 0.87-1.51 | 3.3E-01 | 0.69 |
| **325.19912/2.5748** | 1.01 | 0.92-1.10 | 9.1E-01 | 0.98 | 0.44 | 0.36-0.53 | 1.5E-17 | 1.6E-15 |
| **327.22090/4.1352** | 1.01 | 0.91-1.12 | 9.1E-01 | 0.98 | 0.93 | 0.80-1.09 | 3.8E-01 | 0.72 |
| **313.23813/5.3834** | 0.99 | 0.91-1.09 | 9.1E-01 | 0.98 | 0.98 | 0.83-1.17 | 8.4E-01 | 0.95 |
| **295.22799/5.1623** | 0.99 | 0.89-1.11 | 9.2E-01 | 0.98 | 1.08 | 0.93-1.26 | 3.0E-01 | 0.67 |
| **333.20718/3.9351** | 0.99 | 0.90-1.09 | 9.2E-01 | 0.98 | 1.05 | 0.89-1.24 | 5.6E-01 | 0.82 |
| **287.16729/3.2987** | 1.00 | 0.92-1.07 | 9.2E-01 | 0.98 | 0.88 | 0.70-1.12 | 3.1E-01 | 0.67 |
| **289.18037/3.7720** | 1.00 | 0.93-1.08 | 9.3E-01 | 0.98 | 0.84 | 0.66-1.07 | 1.6E-01 | 0.59 |
| **343.22640/5.767** | 1.00 | 0.91-1.09 | 9.5E-01 | 0.99 | 1.00 | 0.84-1.19 | 9.9E-01 | 1.00 |
| **363.25100/3.1270** | 1.00 | 0.93-1.08 | 9.5E-01 | 0.99 | 0.86 | 0.70-1.06 | 1.6E-01 | 0.59 |
| **327.21641/3.2131** | 1.00 | 0.91-1.10 | 9.6E-01 | 0.99 | 1.03 | 0.86-1.24 | 7.2E-01 | 0.89 |
| **351.21762/4.1352** | 1.00 | 0.89-1.12 | 9.6E-01 | 0.99 | 1.11 | 0.96-1.28 | 1.5E-01 | 0.59 |
| **359.22157/4.8535** | 1.00 | 0.90-1.11 | 9.7E-01 | 0.99 | 1.08 | 0.93-1.26 | 3.2E-01 | 0.69 |
| **395.24273/4.4540** | 1.00 | 0.90-1.10 | 9.8E-01 | 0.99 | 0.98 | 0.83-1.16 | 8.5E-01 | 0.96 |
| **317.21228/4.8006** | 1.00 | 0.89-1.12 | 9.9E-01 | 1.00 | 1.07 | 0.93-1.24 | 3.5E-01 | 0.71 |
| **359.22338/4.4540** | 1.00 | 0.92-1.09 | 1.0E+00 | 1.00 | 1.10 | 0.91-1.32 | 3.2E-01 | 0.69 |
| **327.21761/4.425** | 1.00 | 0.90-1.11 | 1.0E+00 | 1.00 | 0.91 | 0.78-1.05 | 2.0E-01 | 0.60 |

**^#^**Each eicosanoid is represented by MZ/RT; MZ is the mass-to-charge ratio (to 5 decimals) and RT is the retention time (to 4 decimals).

*All models were adjusted for age, sex, height, weight, systolic and diastolic blood pressure, current smoking, use of antihypertensive medication, diabetes, history of myocardial infarction, and history of heart failure

**^†^**HR: hazard ratio expressed per standard deviation of log transformed normalized eicosanoid concentration; OR: odds ration expressed per standard deviation of log transformed normalized eicosanoid concentration; CI: confidence interval

**sTable 3**. Top AF-related eicosanoids with sex-stratified analysis

| **Eicosanoids** | **Men*** | | | **Women only*** | | | **Sex-interaction** |
| --- | --- | --- | --- | --- | --- | --- | --- |
|  | HR^†^ | 95% CI^†^ | P-value**^†^** | HR^†^ | 95% CI^†^ | P-value**^†^** | P-value**^†^** |
| 293.21136/5.1237 | 1.26 | 1.10-1.43 | 6.5x10^-4^ | 1.13 | 0.94-1.34 | 0.18 | 0.28 |
| 299.25921/5.5568 | 1.14 | 1.02-1.29 | 0.03 | 1.17 | 1.02-1.34 | 0.02 | 1.00 |
| 265.17938/3.7720 | 1.23 | 1.08-1.41 | 2.5x10^-3^ | 1.10 | 0.93-1.30 | 0.25 | 0.26 |
| 291.19445/4.3834 | 1.20 | 1.05-1.37 | 7.5x10^-3^ | 1.14 | 0.97-1.33 | 0.12 | 0.55 |
| 321.24360/6.1182 | 1.21 | 1.06-1.38 | 4.0x10^-3^ | 1.10 | 0.94-1.28 | 0.22 | 0.29 |
| 321.24387/5.4933 | 1.19 | 1.04-1.36 | 0.01 | 1.14 | 0.98-1.33 | 0.09 | 0.61 |
| *Covariates include age, smoking, height, weight, systolic blood pressure, diastolic blood pressure, antihypertensive treatment, diabetes mellitus, prevalent myocardial infarction, and prevalent heart failure  **^†^**HR: Hazard ratio, expressed per standard deviation of the normalized eicosanoid concentration; CI: confidence interval; P-values listed here were not adjusted for multiple testing. | | | | | | | |

**sTable 4**. Top AF-related eicosanoids with age-stratified analysis

| **Eicosanoids** | **Older adults**  **(>=65 years of age)*** | | | **Younger adults**  **(<65 years of age) only*** | | | **Age interaction** |
| --- | --- | --- | --- | --- | --- | --- | --- |
|  | HR^†^ | 95% CI^†^ | P-value**^†^** | HR^†^ | 95% CI^†^ | P-value | P-value**^†^** |
| 293.21136/5.1237 | 1.13 | 1.00-1.27 | 0.05 | 1.52 | 1.22-1.89 | 1.9x10^-4^ | 0.02 |
| 299.25921/5.5568 | 1.13 | 1.02-1.25 | 0.02 | 1.29 | 1.09-1.54 | 3.8x10^-3^ | 0.20 |
| 265.17938/3.7720 | 1.18 | 1.05-1.33 | 0.01 | 1.19 | 0.96-1.47 | 0.12 | 0.79 |
| 291.19445/4.3834 | 1.17 | 1.04-1.31 | 0.01 | 1.22 | 0.99-1.51 | 0.07 | 0.59 |
| 321.24360/6.1182 | 1.11 | 1.00-1.25 | 0.06 | 1.35 | 1.10-1.65 | 3.9x10^-3^ | 0.10 |
| 321.24387/5.4933 | 1.08 | 0.97-1.22 | 0.17 | 1.42 | 1.18-1.71 | 1.7x10^-4^ | 0.01 |
| *Covariates include age, sex, smoking, height, weight, systolic blood pressure, diastolic blood pressure, antihypertensive treatment, diabetes mellitus, prevalent myocardial infarction, and prevalent heart failure  **^†^**HR: Hazard ratio, expressed per standard deviation of the normalized eicosanoid concentration; CI: confidence interval; P-values listed here were not adjusted for multiple testing. | | | | | | | |

**sTable 5**. Correlation analysis with CRP and IL-6

| **Eicosanoids^#^** | **Putative identity** | **Correlation coefficient with CRP** | **Correlation coefficient with IL-6** |
| --- | --- | --- | --- |
| 293.21136/5.1237 | 9-oxoODE | 0.13 | 0.12 |
| 299.25921/5.5568 | EIC_33 | 0.04 | 0.09 |
| 265.17938/3.7720 | 12(R) HETE | -0.01 | -0.03 |
| 291.19445/4.3834 | 9-oxoOTrE | -0.03 | -0.04 |
| 321.24360/6.1182 | 15 oxoEDE | 0.09 | 0.07 |
| 321.24387/5.4933 | HETrE [M-H] | 0.04 | 0.06 |
| **^#^**Each eicosanoid is represented by MZ/RT; MZ is the mass-to-charge ratio (to 5 decimals) and RT is the retention time (to 4 decimals).  *Covariates include age, sex, smoking, height, weight, systolic blood pressure, diastolic blood pressure, antihypertensive treatment, diabetes mellitus, prevalent myocardial infarction, and prevalent heart failure  **^†^**HR: Hazard ratio, expressed per standard deviation of the normalized eicosanoid concentration; CI: confidence interval; P-values listed here were not adjusted for multiple testing. | | | |

**sFigure 1.** Survival curves for **293.21136/5.1237.** Lower panel shows the number of participants at risk during the study period.


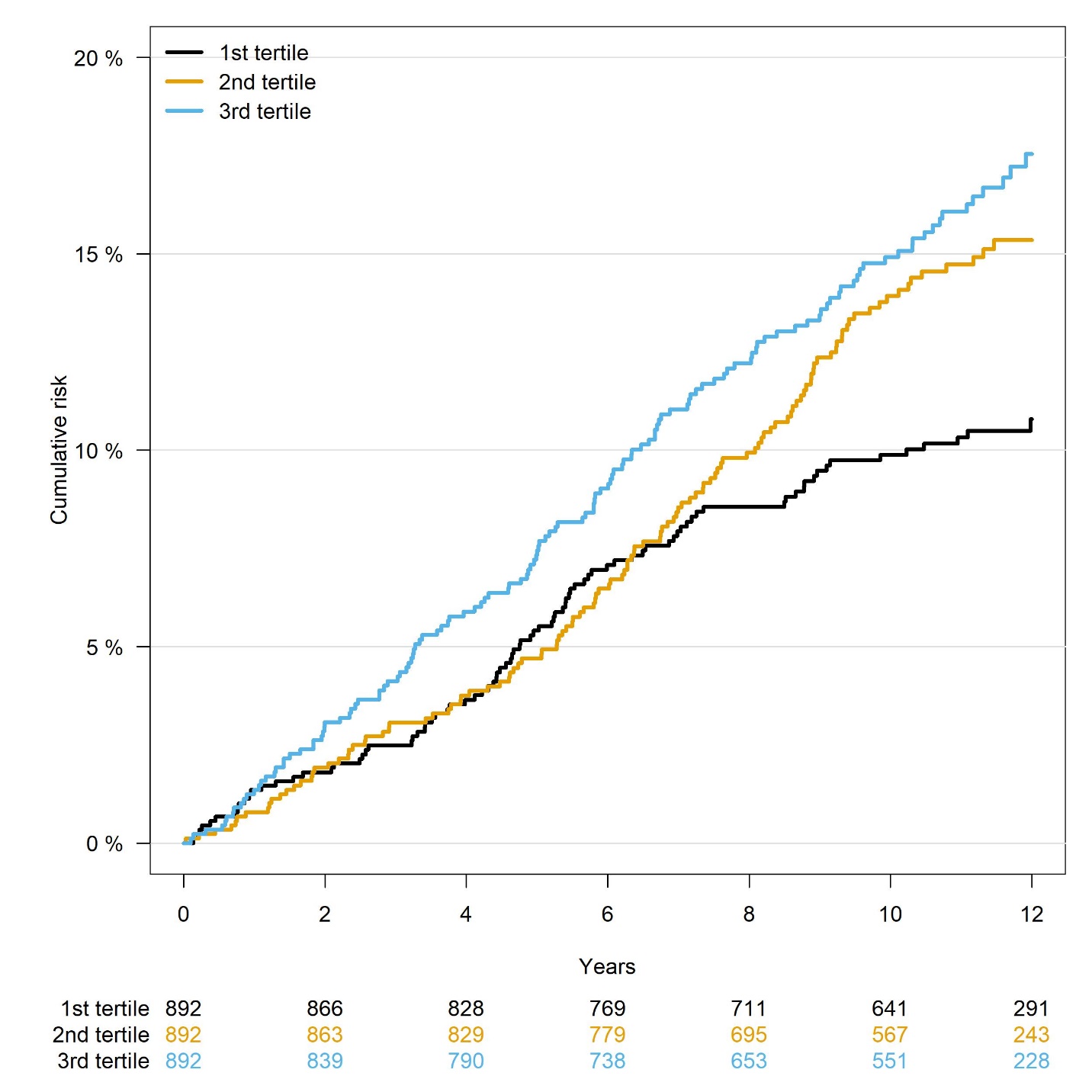


**sFigure 2.** Survival curves for 299.25921/5.5568. Lower panel shows the number of participants at risk during the study period.


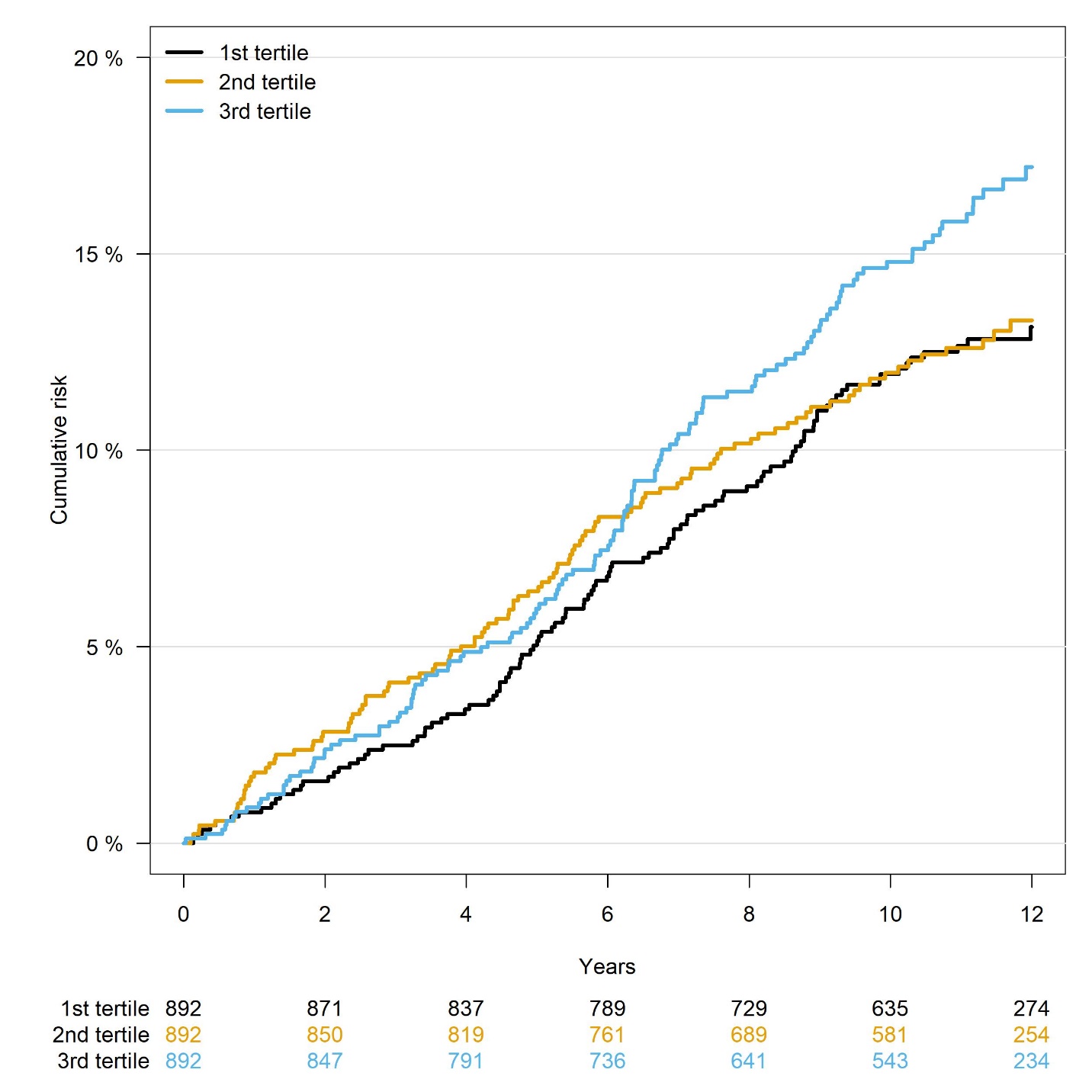


**sFigure 3.** Survival curves for 265.17938/3.7720**.** Lower panel shows the number of participants at risk during the study period.


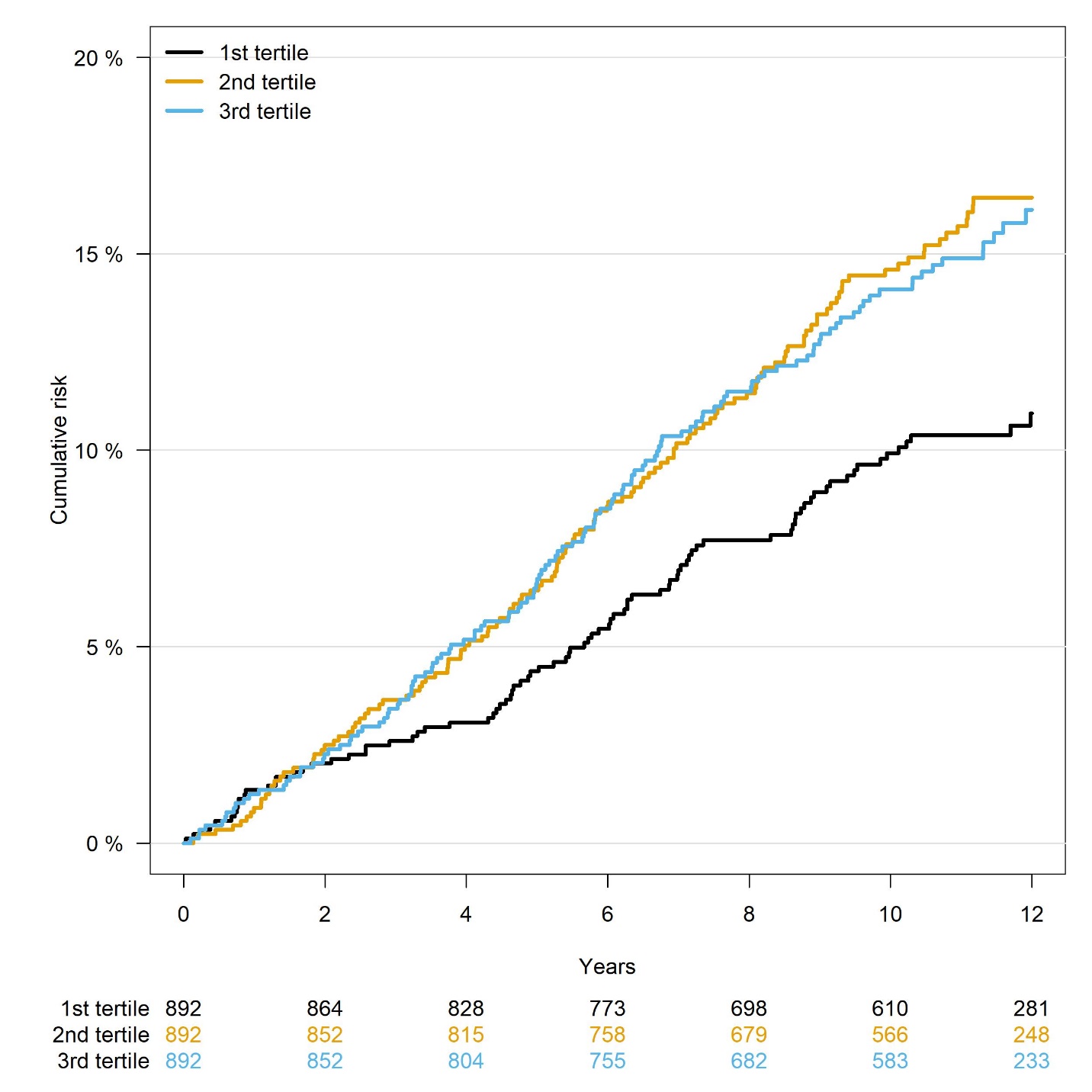


**sFigure 4.** Survival curves for 291.19445/4.3834**.** Lower panel shows the number of participants at risk during the study period.


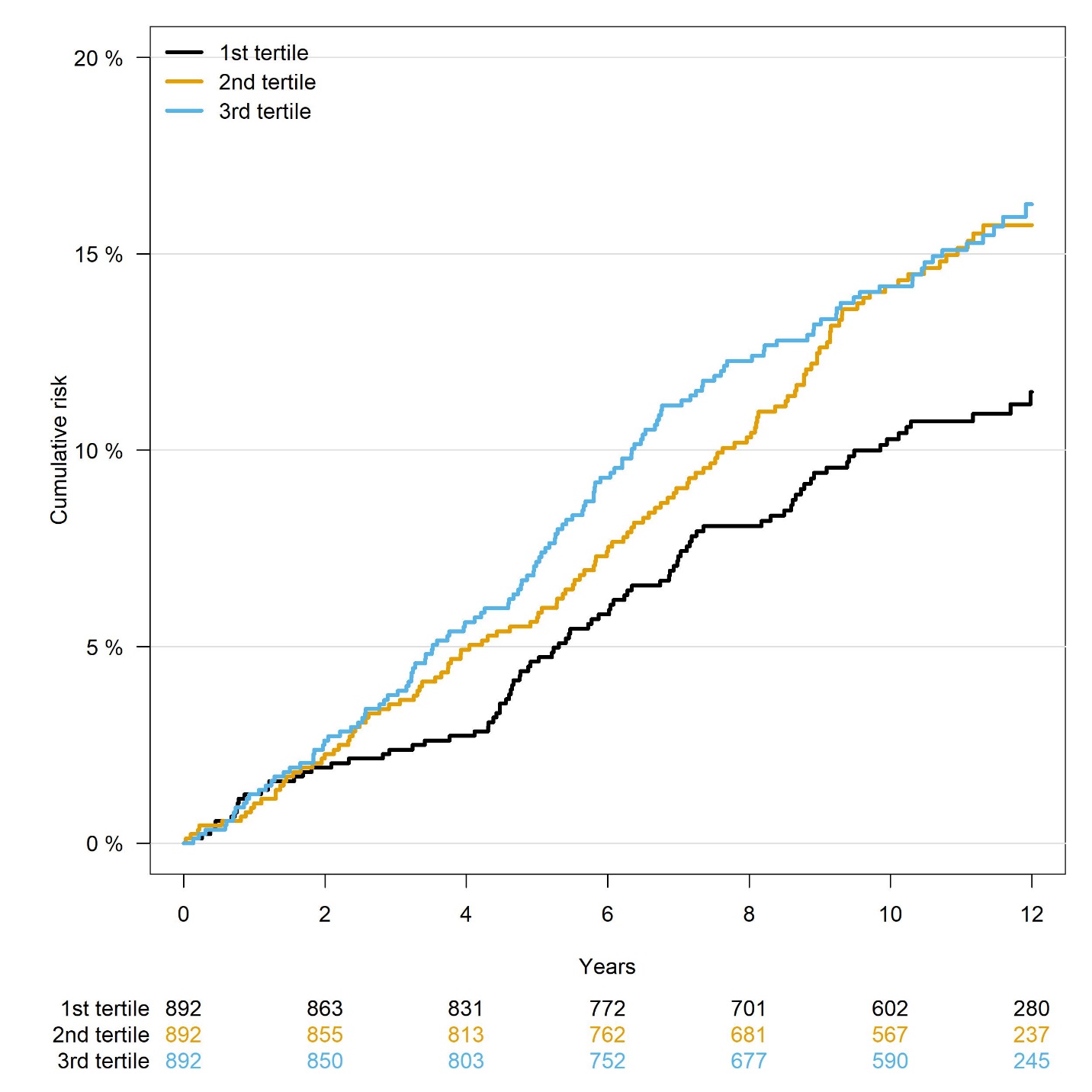


**sFigure 5.** Survival curves for 321.24360/6.1182**.** Lower panel shows the number of participants at risk during the study period.


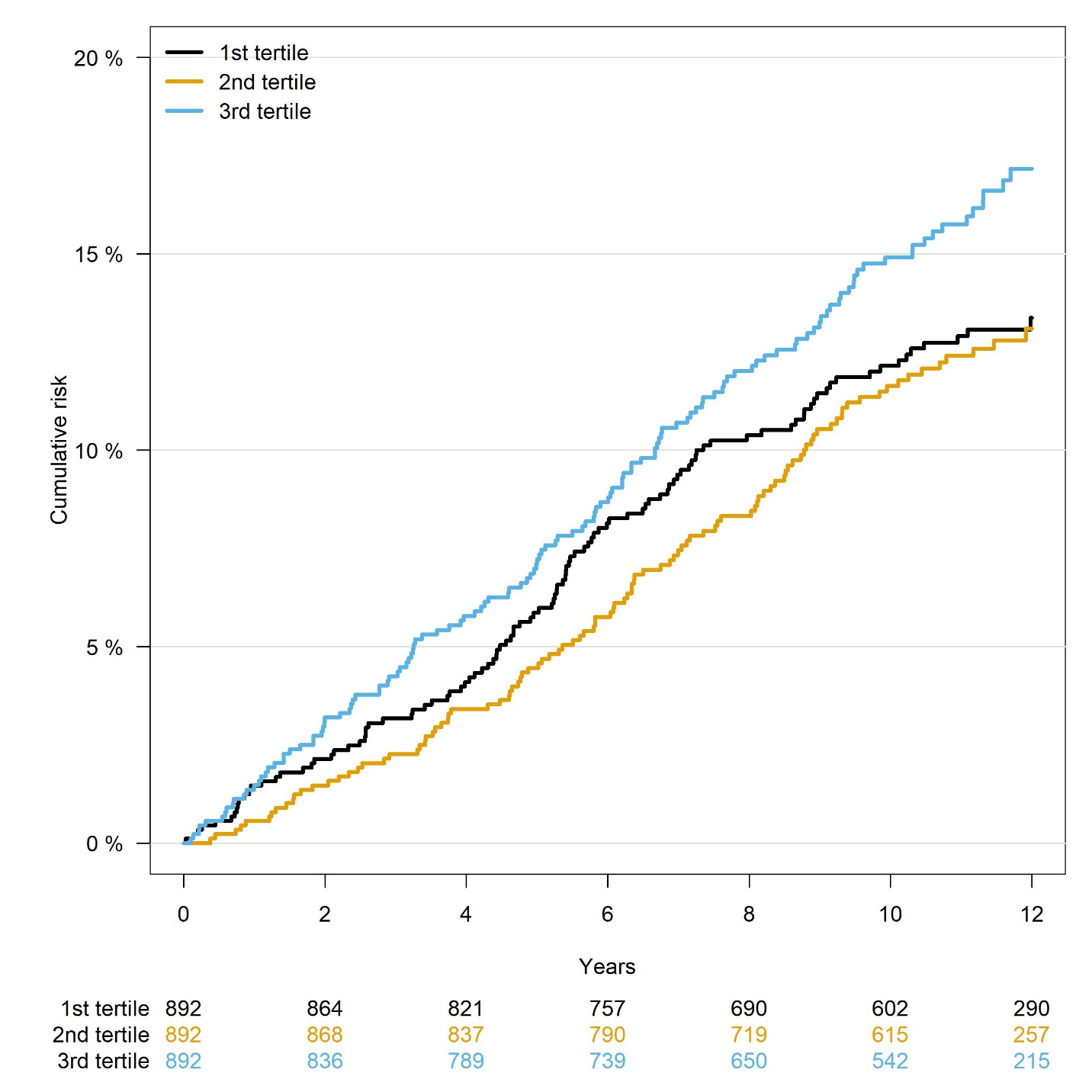


**sFigure 6.** Survival curves for 321.24387/5.4933**.** Lower panel shows the number of participants at risk during the study period.


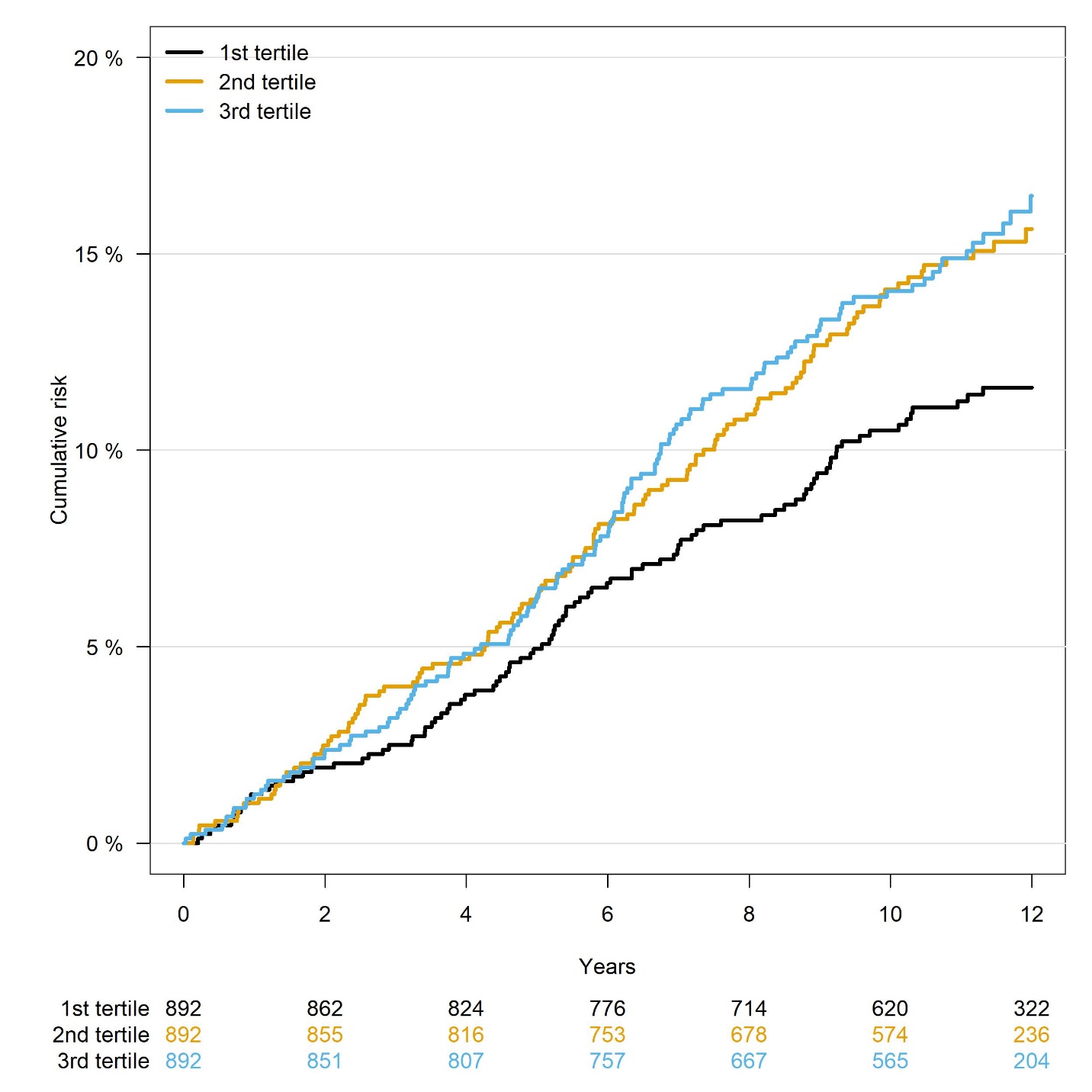

Supplement: Supplementary file 1 — Supplementary Information. [file 41598_2022_21786_MOESM1_ESM.docx]
